# Bond Strength of an Epoxy Resin Root Canal Sealer Prototype

**DOI:** 10.3390/dj13090415

**Published:** 2025-09-09

**Authors:** Matthias J. Roggendorf, Hubert C. Roggendorf, Markus Müller-Krott, Franz-Josef Faber, Roland Frankenberger

**Affiliations:** 1 Clinic for Operative Dentistry, Endodontics, and Pediatric Dentistry, University Dental Medicine, Philipps University Marburg and University Hospital Giessen and Marburg, Campus Marburg, Georg-Voigt-Straße 3, 35039 Marburg, Germany; frankbg@med.uni-marburg.de; 2 Interdisciplinary Department of Oral Surgery and Implantology, University of Cologne, Kerpener Straße 32, 50931 Cologne, Germany; hubert.roggendorf@uk-koeln.de; 3 Center of Dental Medicine, University of Cologne, Kerpener Straße 32, 50931 Cologne, Germany; markus.mueller-krott@uk-koeln.de (M.M.-K.); franz-josef.faber@uk-koeln.de (F.-J.F.)

**Keywords:** AH Plus Jet, endodontics, epoxy resin, obturation, push-out test, root canal sealer

## Abstract

**Background/Objectives**: We aimed to assess the bond strength of AH Plus Jet (AH) and an epoxy resin-based root canal sealer prototype (K-0189) adhered to three different obturation points. **Methods**: A total of 120 single-rooted teeth were selected after radiographic analysis, and their root canals were instrumented with ProTaper Next files (PTN) up to size X5. The teeth were randomly assigned to two sealer groups (G) (G1: AH, G2: K-0189, each n = 60) and further divided into three subgroups: (A) ConformFit X5 points (PTN) cold obturation (CO), (B) ProTaper Universal F5 points (PTU) (CO), (C) GuttaCore X5 (GC) warm obturation (WO). After final irrigation (NaOCl 3%, EDTA 17%, NaOCl 3%) and drying, root canals were obturated and stored for 30 days at 37 °C in Simulated Body Fluid (SBF). The specimens were embedded in acrylate and sectioned horizontally; then, push-out bond strength (POBS) analysis was performed. **Results**: The median POBS values [MPa] were G1A: 2.03; G1B: 2.12; G1C: 3.2; G2A: 1.91; G2B: 2.56; and G2C: 3.36. WO showed significantly higher POBS (*p* < 0.001 *) than CO. The POBS was not significantly different between the two WO groups (*p* = 0.508). The POBS of G2B was significantly higher compared to all other CO groups. **Conclusions**: The epoxy resin sealer prototype demonstrated POBS values comparable to AH Plus when used with WO and PTU points, indicating significantly higher POBS values compared to all other CO points.

## 1. Introduction

Root canal fillings should efficiently seal the instrumented and chemomechanically prepared root canal system to prevent reinfection due to leakage [1]. Due to root canal system access and subsequent chemomechanical preparation, root canal dentin weakens, increasing root dentin defects over time [2,3]. This should be compensated for as much as possible in the root canal filling. Therefore, root canal fillings should be thoroughly adhered to both the obturation point and root canal dentin. Various studies have shown that the use of root canal sealers and obturation techniques can increase teeth’s fracture resistance to a certain extent [4,5]. However, post canal preparation may result in detachment of the remaining root canal filling, which may lead to reduced sealing efficiency [6,7]. In this respect, root canal fillings with higher bond strength are advantageous.

The chemical and mechanical properties of AH Plus allow for high sealing efficiency, adequate volumetric behavior, and significantly lower solubility compared to other sealers [8,9,10,11]. Its suitability for warm and cold obturation techniques is another advantage [12]. Therefore, the epoxy resin sealer AH Plus is the gold standard for root canal sealers as it is well-investigated and widely used [13]. AH Plus shows a significantly higher median dislodgement resistance (7.03 MPa) compared to other sealer materials, such as silicate-based sealers (ranging from 1.6 to 3.5 MPa in 0.04 tapered root canals) [14]. This is due to epoxides forming a covalent bond with the collagen amino groups in the dentin [15]. Calcium silicate sealers, on the other hand, achieve their adhesive bond through the formation of a so-called mineral infiltration zone via increased mineralization, creating tag-like structures [16].

In terms of biocompatibility and bioactivity, epoxy resin sealers reduced cell viability and cell migration [17]. No bioactivity was detected for AH Plus [18]. Calcium silicate sealers have been shown to produce high cell proliferation compared with epoxy resin sealers [19]. This effect is associated with the expression of alkaline phosphatase (ALP), catabolite activator protein (CAP), and cementum protein 1 (CEMP-1) in the presence of silicate sealers, which increases the mineralization capacity [20].

In the present study, we aimed to investigate the dislodgement resistance of root canal fillings with AH Plus Jet in comparison with an epoxy resin root canal sealer prototype (code name: K-0189; both Dentsply DeTrey, Konstanz, Germany) using a standardized measurement procedure via a push-out analysis. Both sealers were used in combination with three different obturation materials, two different types of gutta-percha points, and a thermoplastic obturator. On the one hand, this study determined how an experimental sealer material compares to the gold standard AH Plus with regard to POBS. On the other hand, possible differences between cold and warm obturation were investigated.

The first null hypothesis is that there are no observable differences in POBS between the two sealers. The second null hypothesis is that POBS is not affected by the type of obturation technique.

## 2. Materials and Methods

### 2.1. Preliminary Tests

Preliminary tests were conducted to clarify questions relevant to the actual study regarding the suitability of the methodology. First, the effect of storage time on the bond strength of the sealer prototype was investigated. Secondly, the generation of temperatures during embedding and sectioning was investigated.

#### 2.1.1. Investigation of Storage Time on Bond Strength

For the preliminary study, 20 human teeth were provided by Enretec.dental (Velten, Germany), in compliance with the Declaration of Helsinki [21]. The number of cases was based on a previous pull-out study [22].

The inclusion criteria were defined as follows:

- Single root canal without isthmuses or branches;
- Straight root canal;
- Mature apex;
- No caries or at least the lowest possible degree of destruction;
- Radiographically suitable canal shape;
- Initial canal dimension and shape smaller than the dimension of final file.

The teeth were stored under moist conditions until processing at Enretec and thereafter stored and delivered in 0.5% chloramine solution prior to the preparation of the specimens for disinfection and protection from desiccation. The teeth were examined radiographically (Sirona Heliodent Plus, 80 ms, 65 kV, 7.5 mA, Dentsply Sirona, Bensheim, Germany) to assess their suitability using digital radiographs (VistaScan imaging plate 3 × 4 cm, VistaScan Mini View X-Ray Scanner, Dürr Dental, Bietigheim-Bissingen, Germany).

#### 2.1.2. Preparation of the Access Cavity and Root Canal Preparation

The access cavity was created using cylindric diamond burs (type 6837314014, Komet Dental, Lemgo, Germany). Coronal enlargement of the canal entrance was performed with Gates–Glidden drills # 90 (Komet Dental). Patency of the canals was checked using Patency Files ISO 10 (Komet Dental). The length of the root canals was determined radiographically using calibrated diagnostic X-ray images in combination with an advanced ISO 10 Patency File until the tip of the instrument was visible. Root canals were instrumented with BioRaCe files (FKG Dentaire, La Chaux-de-Fonds, Switzerland) to ISO 60 with an endodontic motor (EndoPilot^2^, Schlumbohm, Brokstedt, Germany). Each instrument was replaced after a maximum of three uses in order to exclude possible wear-related changes in cutting efficiency or instrument fractures. All steps of endodontic treatment were performed under a dental operating microscope (Atmos iView 31 Dental; 20×, Atmos, Lenzkirch, Germany).

Between file changes, irrigation was performed with NaOCl 3% (Speiko, Dr. Speier & Co, Münster, Germany) and repeatedly checked with an ISO 10 Patency File to avoid blockages. For irrigation solutions, Luer Lock syringes (B.Braun, Melsungen, Germany) were color-coded and fitted with VMK Endoneedles (Nipro, Osaka, Japan). A final standardized irrigation protocol was used, consisting of 3 mL NaOCl 3% (Speiko) with activation for 4 × 15 s using EndoActivator (Endo Inventors, Santa Barbara, CA, USA) and EndoActivator Tip (Tianjin Golden Vendor, Tianjin, China) size 0.04/#35 at 9000 oscillations per minute, followed by 3 mL EDTA 17% (Pharmacy of the University Hospital Marburg, Marburg, Germany) and another 3 mL NaOCl 3% (Speiko). Each root canal was then dried with 3 ProTaper NEXT X5 paper points (Dentsply Maillefer, Ballaigues, Switzerland).

Stainless steel spreaders ISO 60 (Komet Dental, Lemgo, Germany) served as obturation points. The spreaders were sandblasted (Rocatec, 3M, St. Paul, MN, USA) prior to obturation. Each root canal was filled with the K-0189 sealer prototype and one steel spreader. Coronal sealer excess was removed with foam pellets (Foam Pellets No. 1, Voco, Cuxhaven, Germany), and the specimens, covered with gauze, were stored for 7 and 30 days (n = 10), respectively. Bond strength was analyzed using a universal testing machine (Zwick 1120 retro line, Zwick Roell, Ulm, Germany) at a cross-head speed of 2 mm per minute, according to the pull-out test of Ebert et al. (2011) [22].

The teeth were wrapped in gauze stripes (Medicomp, Hartmann, Heidenheim, Germany), immediately placed in Eppendorf tubes 2.0 mL (Eppendorf, Hamburg, Germany) filled with SBF [23], and stored vertically in an incubator (Heraeus B 290, Heraeus, Hanau, Germany) at 37 °C to allow for the curing of the root canal sealers for 7 and 30 days, respectively.

In order to assess the homogeneity of the root canal filling and obtain information about the effective length of the root canal filling, X-rays were retaken for all teeth. In order to avoid any influence from horizontal positioning of the specimens, radiographs were taken shortly before the analysis of the specimens at the end of the storage period.

#### 2.1.3. Embedding of the Specimens and Investigation of Generated Temperatures During the Embedding and Cutting Process

To date, no data have been published on the influence of specimen embedding and cutting. Therefore, the resulting temperatures generated during embedding in ClaroCit (Struers, Ballerup, Denmark) and sample cutting were investigated.

**Figure 1:**
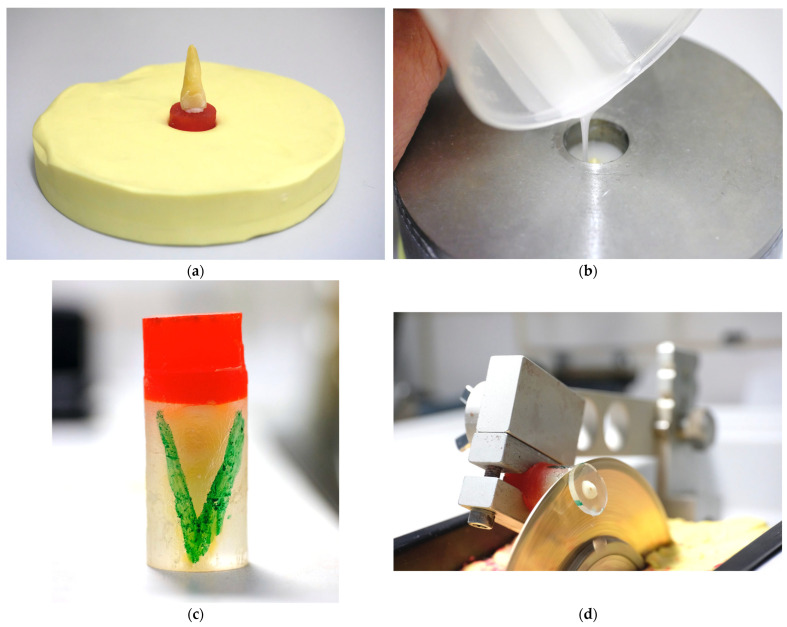
Embedding of the specimens: (**a**) Adjusted tooth on a Palavit G carrier placed in a silicone base (Optosil) after fixing with Venus Diamond composite (Kulzer Dental) and light-curing before embedding in ClaroCit resin. (**b**) Embedding in ClaroCit. (**c**) Specimen after embedding. Above is the Palavit G carrier for safe mounting in the low-speed saw. The transparent ClaroCit embedding contains the tooth and allows good visibility during the trimming process until the apex apparently serving as the zero-position of the cutting blade. The “V” mark allowed for the identification of the correct positioning of the section in the universal testing machine. (**d**) Cutting process immediately before complete separation of the section. Cooling water was permanently delivered by the rotation of the cutting disk.

Teeth were embedded in acrylate resin (ClaroCit) using holed stainless-steel disks (SD Mechatronic, Feldkirch, Germany) with a center hole diameter of 1.6 cm serving as molds. Therefore, the teeth were vertically positioned on individually manufactured carriers (Palavit G, Kulzer Dental, Hanau, Germany) and pre-fixed with resin composite (Venus Pearl, Kulzer Dental). Each carrier was then inserted into prepared silicone plates (Optosil, Kulzer Dental), and the pre-cooled metal mold was positioned on top (Figure 1a). The center hole of the mold was isolated with Vaseline to prevent the Palavit G from sticking to the metal mold and to facilitate removal of the embedded specimen. The ClaroCit embedding material was then poured into the pre-cooled molds (4 °C) (Figure 1b). After curing, the embedded specimens were removed from the metal mold. After apical trimming of the specimens until the root tip was visible, the specimens were labeled on the carrier side with specimen codes and marked with a “V” on both sides of the ClaroCit cylinder for subsequent identification of the correct position in the universal testing machine (Figure 1c). The specimens were mounted in a Buehler IsoMet 1000 precision saw and cross-sectioned with a 15LC saw disk (Buehler, Lake Bluff, IL, USA; Figure 1d).

In order to obtain detailed information about the generated temperatures during the embedding and cutting process, repeated analysis sequences (VarioCAM HD, Jenoptik, Jena, Germany) were carried out using a thermal camera. The temperature development was recorded by means of the IRBIS software 3 plus release 152 (Jenoptik, Jena, Germany) on a connected PC (Dell Technologies Inc., Round Rock, TX, USA).

### 2.2. Main Investigation

A power calculation using G*Power 3.1.9.6 (Heinrich Heine University, Düsseldorf, Germany) with power = 0.8, effect size of 0.25, and level of significance = 0.05 resulted in a sample size of 45 slices per group.

For the main study, another 120 human teeth (Enretec) were identified as suitable when meeting the inclusion criteria described in the preliminary analysis.

#### 2.2.1. Access Cavity, Instrumentation, Irrigation, Activation, and Drying of the Root Canals

The creation of the access cavity and gauging, irrigation, and drying of the root canals was performed similarly to the preliminary tests but using a different instrument system. Root canals in the main investigation were instrumented with ProTaper NEXT (Dentsply Maillefer, Ballaigues, Switzerland) system according to the sequence specified by the manufacturer from X1 to X5 (Table 1).

A total of 60 teeth per sealer group (G1: AH Plus Jet or G2: K-0189) were randomly assigned to 3 subgroups (n = 20): obturation was performed with gutta-percha and sealer (G1 and G2). The content of the sealers is presented in Table 2.

Each sealer was used in combination with three obturation points and techniques, respectively (Table 3). In subgroups A and B, cold obturation (CO) was performed using the cold single-cone technique (SCT). In subgroup C, warm obturation (WO) was performed using the GuttaCore obturators preheated in a Therma-Prep 2 Oven (both Dentsply Tulsa, Johnson City, TN, USA).

#### 2.2.2. Obturation and Sealing of the Access Cavity

Sealer placement was performed in subgroups A and B using EndoActivator with a #35 tip. The sealer was extruded from the double-barrel syringe with an attached Automixing Tip onto a glass plate and picked up with the EndoActivator tip to be placed onto the canal wall by activating the EndoActivator with 10 s of circumferential movements. The procedure was performed twice per canal. A sealer-coated gutta-percha point was then placed in the root canal. In subgroup C, a medium-sized WaveOne paper point (Dentsply Tulsa, Johnson City, TN, USA), slightly coated with sealer, was inserted into the root canal wall and moved circularly inside the root canal. Subsequently, a preheated GuttaCore obturator was inserted into the root canal and held there for 30 s under slight vertical pressure. This was followed by the removal of the applicator handle. After removal of the coronal obturation material, the access cavities were cleaned with foam pellets (Foam Pellets No. 1, Voco, Cuxhaven, Germany) moistened with alcohol and sealed with ChemFil Superior hand-mix cement (Dentsply DeTrey). The teeth were wrapped in gauze strips (Medicomp) and immediately placed in 2.0 mL Eppendorf tubes (Eppendorf) filled with SBF [23] and stored vertically in an incubator (Heraeus B 290) at 37 °C for 4 weeks to allow for curing of the root canal sealers. Control radiographs were taken shortly before the end of the 4-week storage period, as described for the preliminary tests.

#### 2.2.3. Specimen Embedding

After storage of the samples for 30 days under simulated oral conditions, embedding was performed as described in the Section 2.1.

#### 2.2.4. Cutting Process

**Figure 2:**
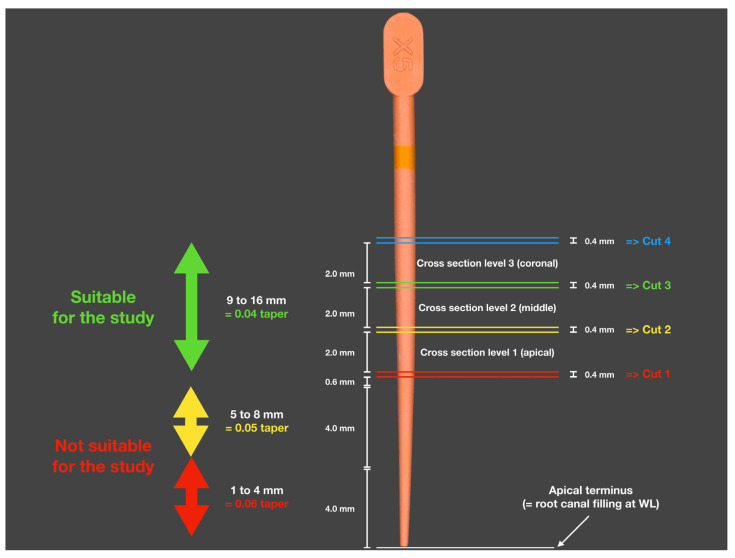
Schematic representation of the section planes for the three cross-sections: 1 = apical, 2 = middle, and 3 = coronal. The colored double lines indicate the cutting loss due to the cutting blade’s thickness of 0.4 mm.

Postoperative control radiographs allowed for the correct positioning of the cutting blade. All three cutting planes per specimen were located within the coronal section of the root canal area with a uniform 0.04 root canal taper (Figure 2).

#### 2.2.5. Analysis of the Diameters of Root Canal Fillings

**Figure 3:**
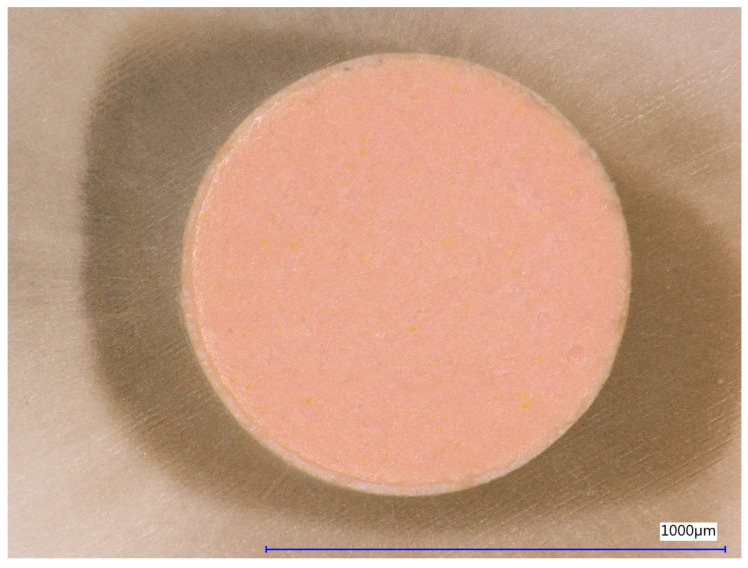
Digital micrograph of the coronal side of a section after obturation with ProTaper X5 ConformFit gutta-percha point and AH Plus Jet sealer.

Prior to the push-out analysis, digital microscopic images (Figure 3) of both sides of the cross-sections were taken using the “Easy Mode” setting (no mapping or focus stacking; Keyence VHX-5000, Keyence Corp., Tokyo, Japan) for subsequent calculation of the effective diameters of the cross-sections. The values determined were recorded in an Excel sheet (Microsoft Corp., Redmond, WA, USA). Based on the analyses of the sections using the following formula, the effective bonding area was individually determined for each section:

$$LSA=\pi (R+r)\sqrt{{\left(R-r\right)}^{2}+h^{2}}$$

Legend: LSA = lateral surface area, π = pi, R = base surface radius, r = top surface radius, and h = thickness of the specimen

#### 2.2.6. Preparation and Implementation of the Bond Strength Analysis

Gates–Glidden drills of various sizes (#50 to #150; Komet Dental) were modified to serve as metal plungers by removing the working part and polishing the sectioning surface. The dimensions of the plunger were analyzed with a digital caliper (Digital Caliper CD-15CPX, Mitutoyo, Kawasaki, Japan), and the appropriate size was selected individually for each sample based on the diameter of the contact surface (Table 4). The exact orthogonality of the separation point to the instrument axis was ensured.

#### 2.2.7. Manufacturing of Customized Holders for Push-Out Analysis

**Figure 4:**
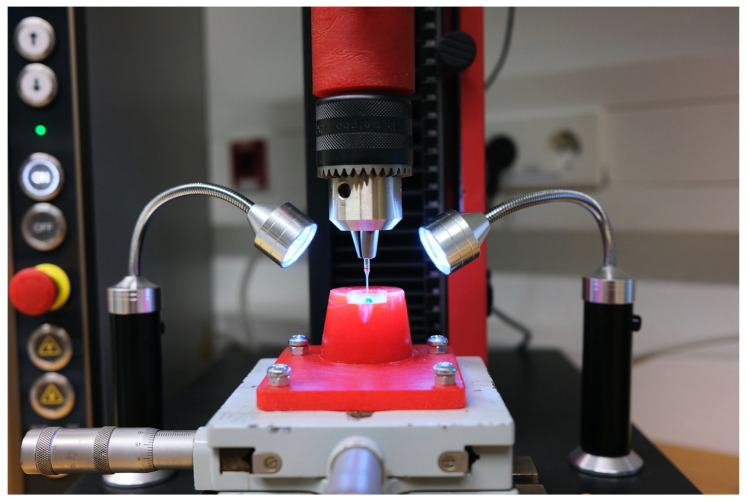
Zwick 1120 universal testing machine with the specimen placed and the tooth slice perfectly centered before the start of the test. On the right and left are two LED lights to ensure adequate adjustment of the slide positioning using the Zeiss cross table.

**Figure 5:**
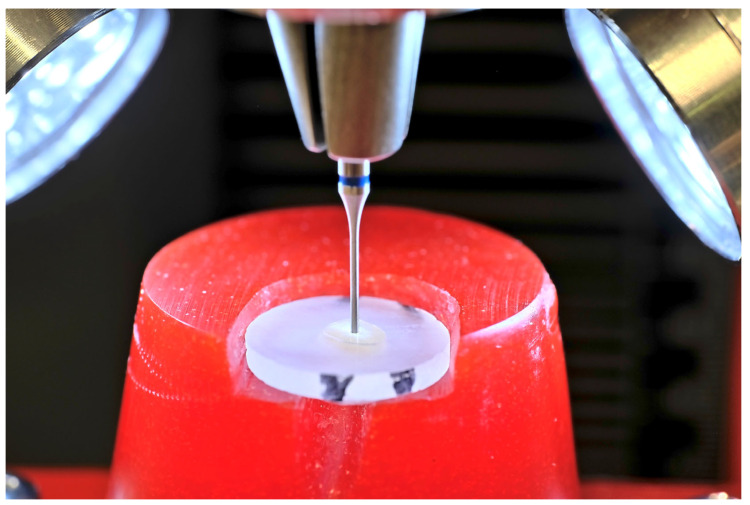
Plunger during the loading of specimen in Zwick 1120 universal testing machine.

The push-out analysis was performed using a Zwick 1120 universal testing machine. The plungers were fixed in a drill chuck (Metabo, Nürtingen, Germany) in the upper holder to enable extrusion of the root canal filling during the push-out analysis. A testing plate with a center hole allowed for the positioning of the sections (Figure 4 and Figure 5). Centering of the sections under the plunger was accomplished using a Zeiss Ikon cross table (Zeiss Ikon, Stuttgart, Germany) equipped with micrometer screws for adjustments in the X and Y axes. The centering of the plunger on the root canal filling was carried out using 2.5× magnifying glasses (Orascoptic, Madison, WA, USA) and two LED lights.

The specimens were placed in the lower fixture of the Zwick 1120 universal testing machine. Based on analysis of previously taken photos of the cross-sections, the required plunger diameter was determined, and the appropriate plunger was selected. The plungers were vertically moved against the root canal filling with a cross-head speed of 1 mm per minute, controlled by testXpert III software Version 1.6 (Zwick Roell). The push-out force [N] determined in this process was calculated and recorded using the software. The resulting maximum force F_max_ [N] of the respective specimen was converted according to the existing canal surface to allow for the expression of the data in MPa.

#### 2.2.8. Performing Fractographic Analysis

The extruded root canal fillings, as well as the sections, were returned to the coin capsules after analysis and later evaluated microscopically to determine the fracture mode. For this purpose, both the tooth section in the area of the root canal and the ejected root canal filling were examined under a digital microscope at 50× magnification (Keyence VHX 5000), and the determined fracture mode was entered in a table. Four fracture modes were possible to rate (Table 5).

#### 2.2.9. Statistical Evaluation

After the analysis, statistical evaluation of the data was performed using SPSS 28 (IBM Corp., Armonk, NY, USA). In addition to descriptive analysis, an analysis for normal distribution of the values was performed using the Shapiro–Wilk test. Since normal distribution and variance homogeneity were not present in all cases, further analyses were performed using non-parametric test procedures (the Kruskal–Wallis and Mann–Whitney tests). The correlation between bond strength and fracture mode, as well as the distribution of fracture modes between the experimental groups, was calculated with the X^2^ test.

## 3. Results

### 3.1. Preliminary Study Results

#### 3.1.1. Effect of Storage Time on Pull-Out Bond Strength

The median pull-out bond strength values in 0.02-tapered root canals revealed a significant increase after 30 days compared to 7 days of storage (7 days: 6.49 MPa, 30 days: 9.48 MPa; *t*-test: *p* = 0.002 *).

#### 3.1.2. Temperatures Generated During Embedding and Sectioning of the Specimens

**Figure 6:**
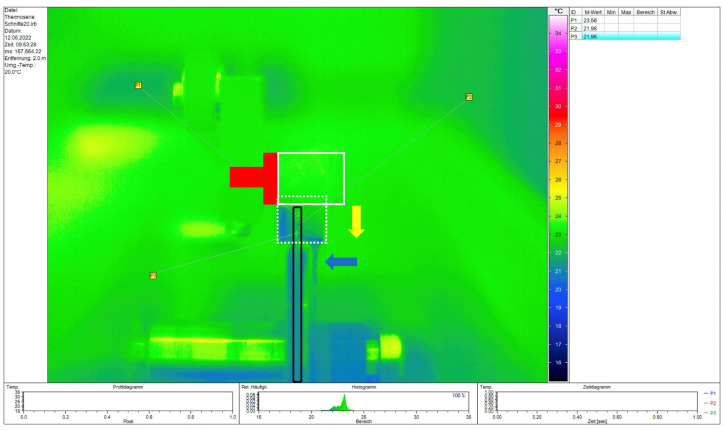
Plot of the determined temperatures during the cutting process under permanent water-cooling of an embedded specimen in the low-speed saw in the presented table shows values below 24 °C. Red T = Palavit G carrier; white rectangle = ClaroCit resin containing the embedded tooth before cutting; black rectangle = cutting blade; yellow arrow = movement of the specimen during cutting. White dotted rectangle = position of the embedded specimen shortly before section was finished. P1 to P3 = generated temperatures during the cutting process. Blue arrow = cooling water dropping down off the embedding block.

The maximum temperature observed during the embedding process was recorded, and the setting of the resin never exceeded 34 °C when pre-cooled stainless-steel disks were used for embedding. Sectioning of the embedded specimens was performed using a Buehler IsoMet 1000 precision saw with a 15LC saw disk (Buehler). The resulting temperatures were always below 24 °C, which showed that the cutting process did not result in the generation of critical temperatures (Figure 6). Both temperatures were rated as safe for the processing of the specimens.

### 3.2. Main Study Results

**Figure 7:**
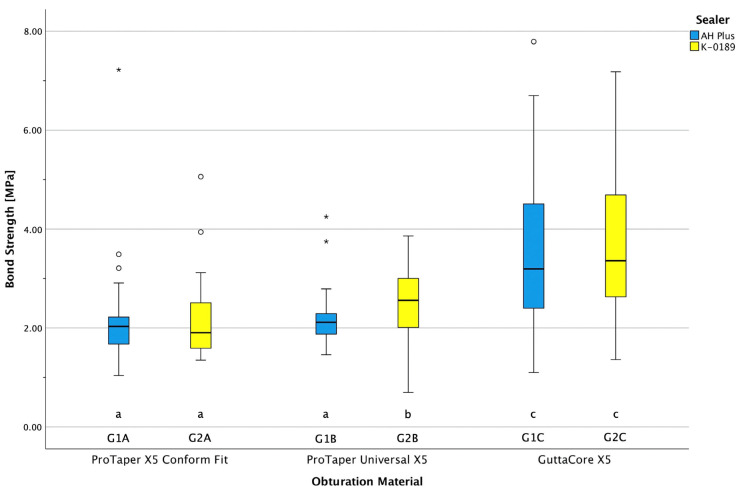
POBS values of the two sealers, AH Plus and K-0189. Different letters (a, b, or c) indicate significant differences between the groups (*p* ≤ 0.05). ○ = mild outlier, * = extreme outlier.

The descriptive data analysis of the push-out values for AH Plus Jet and K-0189 is presented in Table 6. Significant differences between the subgroups are shown in Figure 7. Three out of six groups revealed no normal distribution (the Shapiro–Wilk test, *p* < 0.05 *).

### 3.3. Non-Parametric Analysis of the Bond Strength Values

The Levene statistics showed that there was no homogeneity of variance. Furthermore, the group strengths were not identical in some cases because, in some samples, the levels could not be evaluated, such as when the root canal was too short to obtain three measurements from the sample (one per level). In such cases, non-parametric analysis should, therefore, be used. To analyze possible significant differences between all groups, non-parametric analysis was conducted using the Kruskal–Wallis test as a rank sum test and the Mann–Whitney U test for pairwise comparisons. The non-parametric analysis showed that in the cold obturation groups, bond strength values differed significantly in two pairs of groups (indicated with a *; Table 7). In contrast, there were no significant differences in POBS within subgroup A (ProTaper X5 Conform Fit). The POBS values were not significantly different between the two WO groups after using the two sealers (*p* = 0.508). The POBS values of WO with both sealers were significantly higher than those in the four CO groups (Table 7).

### 3.4. Fractographic Analysis

**Figure 8:**
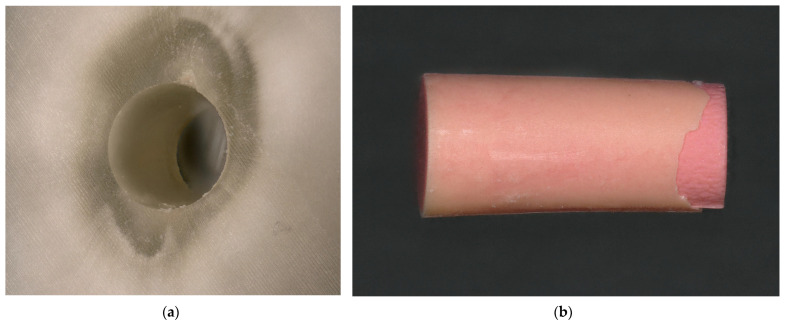
Adhesive fracture to the dentin. (**a**) The root canal surface is completely free of root canal sealer; (**b**) the corresponding gutta-percha point is almost completely covered with an even layer of sealer.

**Figure 9:**
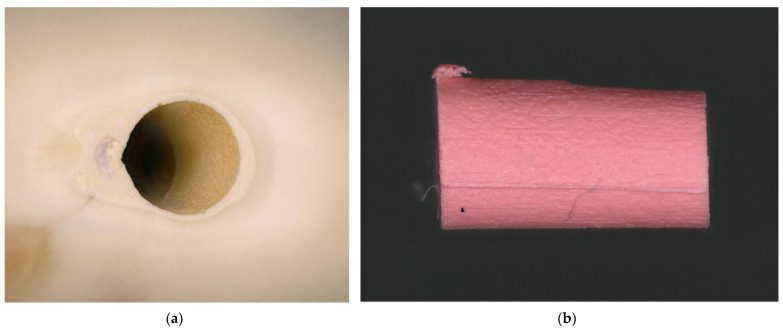
Adhesive fracture to the gutta-percha point. (**a**) The root canal surface is completely covered by a sealer layer; (**b**) the surface of the corresponding gutta-percha point shows no root canal sealer on its surface.

**Figure 10:**
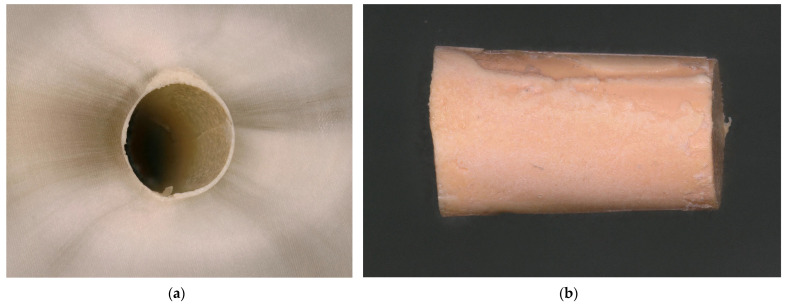
Cohesive fracture. (**a**) A thin layer of sealer is visible on the root canal surface; (**b**) a predominantly thin sealer layer can be seen on the surface of the gutta-percha point, which, however, appears less clearly due to the lower color contrast of both materials.

**Figure 11:**
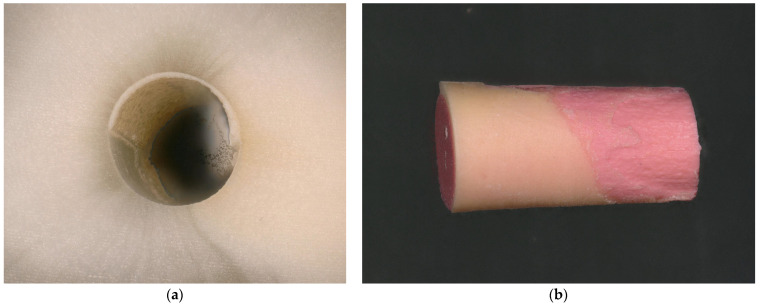
Mixed fracture mode. (**a**) A partially uniform sealer-covered portion of the canal surface can be seen. However, sealer-free canal sections can also be seen, so no predominant fracture mode could be determined in the overall analysis of the specimen; (**b**) the two fracture modes, “adhesive fracture to the gutta-percha post” and “adhesive fracture to the dentin”, as well as smaller areas with recognizable cohesive fracture, led to this assessment.

All sections and root canal fillings were inspected visually using the Keyence VHX-5000 digital microscope. It was possible to choose between four different fracture modes (Table 5). Fracture modes were recorded, and representative examples for each mode were captured using the mapping and focus stacking function of the digital microscope at 200× magnification and assembled automatically. The appearance of both the horizontal sections and the root canal fillings was determined. After complete examination of the samples, the predominant fracture mode was documented for each horizontal cut. Representative examples of the four fracture modes are shown in Figure 8, Figure 9, Figure 10 and Figure 11.

### 3.5. Evaluation of Fracture Modes

Only in the analysis of one specimen an adhesive fracture mode to the dentin was recognizable (Table 8). The cold obturation groups predominantly showed adhesive fractures to the gutta-percha post, whereas this fracture mode was only rarely or not at all observed with GuttaCore. Cohesive fractures, on the other hand, were hardly observed with cold-filling techniques, but were more frequently observed with warm-filling techniques (AH Plus/GuttaCore: 38%) and, in some cases, were even predominant (K-0189/GuttaCore: 78.04%). The mixed fracture mode was the dominant fracture mode in AH Plus, both in the cold filling group with ProTaper X5 points and in the AH Plus/GuttaCore group. Specimens with higher bond strength values predominantly exhibited cohesive or mixed loss modes. Statistical analysis revealed no correlation between bond strength value and fracture mode (X^2^ test: *p* = 0.136). The distribution of the fracture mode revealed significant differences between the obturation groups (X^2^ test: *p* < 0.001 *).

## 4. Discussion

### 4.1. Method

#### 4.1.1. Storage Period and Storage Medium

The preliminary tests showed significantly higher bond strength values after 30 days of storage compared to 7 days. Higher POBS was found after 3 months of storage, compared to 2 weeks, although the difference was not significant for AH Plus [24]. Another study found lower POBS, with values ranging from 1.7 to 1.8 MPa, after 7 days of storage [25]. Based on this study and the preliminary results, all specimens for this study were stored for 4 weeks to achieve maximum bond strength. In another study, simulated root canals with parallel canal walls were obturated with only AH Plus sealer and stored in phosphate-buffered saline for 7 and 30 days, respectively. The results showed very high POBS values (median 13.7 MPa) [26]. To simulate the clinical situation as realistically as possible, SBF was used as the storage medium [23].

#### 4.1.2. Analysis of Temperatures During Embedding and Cutting

Despite a known temperature rise in resins due to the exothermic reaction during polymerization, little information on it is available in the literature. To avoid affecting the root canal filling, the polymerization temperature should be kept as low as possible. The pre-cooled stainless-steel disks ensured a low polymerization temperature (below 30 °C) and were rated as safe, without any expected negative effect on the root canal filling. The potential temperature increase was investigated using the precision saw; it resulted in maximum temperatures of 24 °C, which were rated as not critical for the sectioning of the specimens. The temperature generated between the cutting blade and the specimen may reveal potentially higher values than when analyzed by thermography, but the effect of permanent water cooling seems suitable to keep the generated maximum temperature below body temperature, as no detachments or deformations were detected in digital microscope images prior to POBS analysis. However, sectioning using low-speed saws has been used in several other push-out studies [27,28,29]. Although the thermographic analysis used in this study did not allow for recording the temperature at the section surface, the use of a high-sensitivity thermographic device, as well as the positioning, enables reliable measurement of the generated temperature to rate the sectioning method as safe for this purpose.

#### 4.1.3. Irrigation Protocol and Drying of the Root Canals

The chemomechanical instrumentation is established in endodontic treatment due to its efficiency in reducing bacterial load in the root canal system [30]. NaOCl and EDTA are indispensable in modern endodontics, as they effectively remove the smear layer [31]. The effect of smear layer removal has been rated differently. While some studies did not reveal improved bond strength for AH Plus [25,26], a systematic review showed advantages of using chelators and an associated increase in bond strength [32]. The removal of both organic and inorganic components can be performed using NaOCl and EDTA [30]. A final irrigation with NaOCl after removal of the smear layer by EDTA was beneficial and allowed for the removal of organic deposits and components within the dentin tubules, using the final irrigation described in NaOCl studies [33]. The activation of endodontic irrigants using EndoActivator leaves no smear layer, machining, or defects on the root canal surface and cleans the root canal surface efficiently [34,35]. Sufficient drying of the root canals with this procedure was also described in the literature and could improve the adhesive bond of different root canal sealers [36]. Although moisture did not affect the POBS of AH Plus [28], it was a crucial factor for the sealing efficiency [37].

#### 4.1.4. Selection of the Cutting Planes

The design of the ProTaper NEXT instruments results in different tapers within the prepared root canal. In order to exclude the influence of different tapers, all sections were taken from the coronal 0.04-tapered canal region. The POBS of different canal levels in the present study did not show significant differences between the different canal levels. These findings are in agreement with other studies that reported similar results and showed that the canal level had no significant effect on the POBS when there was a uniform root canal taper [14,38].

#### 4.1.5. Bond Strength Analysis

The push-out method is a well-established method for bond strength analysis of root canal sealers and enables good comparability of the data with results from other studies [39,40,41]. In addition, this technique allows for the comparison of bond strength values between different canal regions [42]. However, no uniform test parameters exist with regard to the thickness of the slices. Push-out tests use slices of 1 mm [25,28,29,43] or 2 mm [24,38,44,45]. We chose the classic push-out test because the thicker section is potentially less affected by factors like the rotation of the cutting blade, cooling water, and surface temperature rise at the cutting surface. Furthermore, the cross-head speeds vary from 0.5 mm/min [25,29,44] to 1 mm/min [27,46]. There is no information on the effect of the test speed on the POBS results. The plunger diameter was found to be potentially relevant for the POBS [47]. Therefore, the plunger should cover at least 90% of the tested root canal filling area in order to obtain reliable analysis results. In contrast, loading only 50–60% of the root canal filling had a negative effect on the adhesive bond determined. Nagas et al. (2011) previously determined a significant influence of the impact diameter on the adhesive bond of root canal sealers, including AH Plus [47].

#### 4.1.6. Limitations of the Study

The first limitation is the analysis of the investigation of generated temperatures. The recorded temperature can be affected by different factors that potentially resulted in a lower temperature than was effectively present. Analyzing the effective temperature at the core of the tooth embedding and in the area of the cutting disk’s surface would require a very intensive analysis, which was not the aim of this investigation. The aim here was to use preliminary tests to examine the suitability of the temperatures displayed using thermography to determine whether temperatures could already be identified that would have required an alternative approach. The second limitation is the lack of information on tooth age. This may have resulted in potentially uneven age distributions in the random assignment of teeth to the different groups. The third limitation relates to the cutting process. The rotation of the cutting disk may have caused vibrations or deformations at the cuts and potentially influenced the POBS values. Possible effects are suspected but have not yet been described in detail in the literature. The fourth limitation is the fact that only epoxy resin sealers were examined. A comparison with silicate-based sealers was not carried out, so no comparison with another material class is possible. However, we intended to compare the POBS of an established epoxy resin sealer and a prototype sealer in combination with two different CO points and a WO technique.

### 4.2. Discussion of Results

#### POBS Values and Comparison with Other Studies

POBS was slightly higher in some cases for the prototype sealer K-0189. In addition, the POBS values for the WO groups (subgroup C) were the highest compared to the three obturation groups in this analysis. These findings are in agreement with the data of Horiuchi et al. (2016), who used root canals instrumented with ProTaper Universal F5 and achieved higher bond values with WO (range from 2.68 to 2.96 MPa) [42]. Other studies under similar conditions also found significantly higher POBS for AH Plus compared to other sealer formulations [38]. The study by El-Ma’aita et al. (2013) with root canal size of taper 0.05/#50 revealed slightly lower POBS values (ranging from 1.98 to 2.09 MPa) for AH Plus than those in the present study [48]. Another study under rather comparable conditions (ProTaper F5 instrumentation, slices of 2 mm thickness) revealed significantly lower POBS for single-cone technique obturation (0.78 MPa) compared with the present study [49].

Taper has a decisive influence on the bond strength of root canal fillings. The POBS values measured in this study are a result of shear and pull tests, which are common in the POBS values obtained from tapered root canals. Simulated cylindric root canals showed POBS between 8 and 13.7 MPa compared to other studies and the results of this study [26,50]. Although cylindric root canals are clinically relevant because most root canal instruments result in tapered preparations, these POBS values demonstrate the bonding efficiency of AH Plus when the influence of “taper” is excluded.

The significantly higher adhesion values of the WO subgroups could have various causes. On the one hand, insertion of the GC obturator causes a temperature increase, which likely causes a reduction in the viscosity of both sealers, as described for epoxy resin sealers [51]. In addition, the plasticization of the gutta-percha allows the obturator to deform during insertion into the root canal. This improves adaptation to the canal wall and increases the flow of the epoxy resin sealer AH Plus, enabling deeper penetration into the dentinal tubules [52,53]. Compared to the relatively smooth gutta-percha points of the CO subgroups, this may have increased the retention area, which is likely to have increased the adhesive bond. The combination of the described effects of temperature increase on epoxy resin sealers seems to be responsible for the significantly higher adhesive bond of both sealers during warm obturation.

Within the limitations of this study, the POBS values of K-0189 were at least similar to, and in some cases even superior to, AH Plus. The POBS values in this study were comparable or slightly higher than those in other studies also using 0.04-tapered root canals [25,27]. This study revealed that the new epoxy resin sealer prototype achieves POBS values that are at least comparable to or superior to those of AH Plus.

There were significant differences between the two sealers detected in subgroup B, with significantly higher POBS for K-0189. Furthermore, WO revealed significantly higher POBS than all CO groups. Therefore, both null hypotheses were rejected.

## 5. Conclusions

Both sealers achieved significantly higher and similar POBS with WO techniques. In addition, K-0189 with ProTaper Universal Point showed significantly higher bond strength values compared to both AH Plus CO groups. POBS of K-0189 with ProTaper NEXT was not significantly different from that of the two AH Plus CO groups.

## Figures and Tables

**Table 1:** Instrument sequence used for root canal instrumentation.

Instrument	Taper/Size	LOT
ProGlider ProTaper NEXT X1 ProTaper NEXT X2 ProTaper NEXT X3 ProTaper NEXT X4 ProTaper NEXT X5	0.02/#16	1715532
	0.04/#17	1717171
	0.06/#25	1720579
	0.07/#30	1717170
	0.06/#40	1466954
	0.06/#50	1721076

**Table 2:** Content of AH Plus Jet and experimental sealer formulation K-0189.

Sealer	Paste A	Paste B
Group 1: AH Plus Jet (LOT: #2201001033)	Calcium tungstate, epoxy resins, zirconium oxide, polydimethylsiloxane, silicon dioxide, and iron oxide pigments.	Calcium tungstate, zirconium oxide, amine resins, silicon dioxide, and polydimethylsiloxane.
Group 2: K-0189 (LOT: #2201004013) ^1^	Calcium tungstate, epoxy resins, silicon dioxide, polymeric additives, and iron oxide pigments.	Calcium tungstate, amine resins, silicon dioxide, polydimethylsiloxane, and polymeric additive.

^1^ Sealer formulation was still a prototype at the start of the study.

**Table 3:** Subgroups representing the different obturation materials.

Obturation Point	Obturation Technique	LOT	Manufacturer
**A:** ProTaper NEXT X5 gutta-percha point	Cold Obturation (CO)	0000294451	Dentsply Maillefer
**B:** ProTaper Universal F5 gutta-percha point	Cold Obturation (CO)	384061O	Dentsply Maillefer
**C:** GuttaCore X5 obturator	Warm Obturation (WO)	0000340472	Dentsply Tulsa

**Table 4:** Plunger sizes after modification of Gates–Glidden burs by removing the tip.

Gates–Glidden Bur	Size	Diameter of Plunger [mm]
white	50	0.35
yellow	70	0.45
red	90	0.55
blue	110	0.65
green	130	0.75
black	150	0.85

**Table 5:** Description of the fracture modes.

Fracture Mode	Visual Appearance
adhesive to dentin	The root canal surface was largely or completely free of root canal sealer or gutta-percha, while the gutta-percha was largely covered with sealer.
adhesive to gutta-percha	The root canal filling was largely uncovered by sealer, but there was a visible layer of sealer on most of the root canal surface.
cohesive	A thin sealer film covered both the canal surface and the gutta-percha filling.
mixed	Two or more fracture patterns were identified, but no clearly predominant fracture pattern could be determined.

**Table 6:** Descriptive data of the statistical analysis [MPa].

Group	Obturation	Mean	SD	Median	Min.	Max.	IQ	*p*-Value ^a^
1A	AH/PTX	2.09	0.95	2.03	1.04	7.22	0.57	<0.001 *
1B	AH/PTU	2.16	0.52	2.12	1.46	4.25	0.45	<0.001 *
1C	AH/GC	3.49	1.50	3.20	1.10	7.79	2.14	0.058
2A	K/PTX	2.19	0.89	1.91	1.35	5.06	0.96	<0.001 *
2B	K/PTU	2.52	0.73	2.56	0.70	3.86	1.02	0.314
2C	K/GC	3.65	1.44	3.36	1.36	7.18	2.21	0.478

Legend: SD = standard deviation; Min. = minimum; Max. = maximum; IQ = interquartile (a = the Shapiro–Wilk test, * = significant).

**Table 7:** Non-parametric analysis of the bond results.

		1A	1B	1C	2A	2B	2C
	Sealer/Cone	AH/PTX	AH/PTU	AH/GC	K/PTX	K/PTU	K/GC
1A	AH/PTX	-	0.155	<0.001 *	0.927	<0.001 *	<0.001 *
1B	AH/PTU	0.155	-	<0.001 *	0.223	0.001 *	<0.001 *
1C	AH/GC	<0.001 *	<0.001 *	-	<0.001 *	<0.001 *	0.508
2A	K/PTX	0.927	0.223	<0.001 *	-	0.011 *	<0.001 *
2B	K/PTU	<0.001 *	0.001 *	<0.001 *	0.011 *	-	<0.001 *
2C	K/GC	<0.001 *	<0.001 *	0.508	<0.001 *	<0.001 *	-

Legend: AH = AH Plus; K = K-0189; GC = GuttaCore; PTX = ProTaper NEXT point; PTU = ProTaper Universal point (* = significant).

**Table 8:** Percentage of the fracture modes in the experimental groups.

Group	Adhesive to Dentin	Adhesive to Gutta-Percha	Cohesive	Mixed
1A	0	34.88	2.33	62.79
1B	0	72.73	2.27	25.0
1C	0	0	38.0	62.0
2A	0	91.67	0	8.33
2B	1.82	70.91	3.64	23.63
2C	0	12.20	78.04	9.76

## Data Availability

Reasonable requests for the presented data may be directed to the corresponding author.
